# PacBio Full-Length Transcriptome Sequencing Reveals the Mechanism of Salt Stress Response in *Sonneratia apetala*

**DOI:** 10.3390/plants12223849

**Published:** 2023-11-14

**Authors:** Beibei Chen, Tingting Liu, Zhuanying Yang, Shaoxia Yang, Jinhui Chen

**Affiliations:** 1 Mangrove Research Center of Guangdong Ocean University, College of Coastal Agricultural Science, Guangdong Ocean University, Zhanjiang 524088, China; beibeichenk@outlook.com (B.C.); liutingting231@mails.ucas.ac.cn (T.L.); Irene0411@aliyun.com (Z.Y.); yangxx@gdou.edu.cn (S.Y.); 2 School of Breeding and Multiplication (Sanya Institute of Breeding and Multiplication), Hainan University, Sanya 572019, China

**Keywords:** *Sonneratia apetala*, salt stress, full-length transcriptome, transcriptional regulation

## Abstract

*Sonneratia apetala* is an essential mangrove wetland restoration tree species. Studying its molecular mechanism for salt tolerance could lay a foundation for further cultivating excellent resistant germplasm. This study used a combination of PacBio isoform sequencing (Iso-seq) and BGISEQ RNA sequencing (RNA-seq) to analyze the molecular mechanism to salt stress response of one-year-old *S. apetala* leaves. The growth and physiological analysis showed that physiological indexes such as growth rate, net photosynthetic rate and antioxidant enzyme activity all exhibit significant changes under salt stress. From Iso-seq, a total of 295,501 full-length transcripts, with an average length of 1418 bp, were obtained. RNA-seq produced 4712 differentially expressed genes (DEGs) as compared to a control group. Of these, 930 were identified to be co-expressed during the STEM time sequence analysis. Further, 715 and 444 co-expressed DEGs were annotated by GO and KEGG analyses, respectively. Moreover, 318 of the co-expressed DEGs were annotated as essential genes that were implicated in salt stress response of *S. apetala*, which were involved in transcription factors, signal transduction, hormone response, ROS homeostasis, osmotic balance, cell wall synthesis or modification. These results provide candidate targets for further characterization and offer insights into the salt-tolerant mechanism of *S. apetala*.

## 1. Introduction

Soil salinization presents a significant challenge to contemporary agriculture and forestry. Elevated salt levels not only decrease soil fertility and productivity but also exert deleterious effects on plant growth and development [1]. Specifically, high salt concentrations induce osmotic stress in the cells, which results in a reduction in water utilization efficiency, disrupted metabolic processes and, ultimately, inhibited photosynthesis, which finally culminates in a plant’s death [2]. Leaves are the primary site of photosynthesis and play a critical role in a plant’s growth and development [3]. Therefore, investigating the response of plant leaves to salt stress is helpful in elucidating the underlying mechanisms against salt stress tolerance.

Based on their sensitivity to salt stress, plants can be classified into halophytes and glycophytes [4]. Glycophytes are highly salt-sensitive plants; typically, they are intolerant. In contrast, halophytes are tolerant and can grow normally under high salt concentrations. Halophytes can be further classified into true, salt-secreting and facultative halophytes. Halophytes possess innate mechanisms of salt tolerance, enabling them to respond rapidly to salt stress, thereby serving as excellent natural materials for salt-resistance breeding [5].

When plants are subjected to salt stress, they can reduce the damage caused to their cells by promoting selective absorption of inorganic ions, synthesis of regulatory substances such as hormones and increased antioxidant enzyme activity. The initial response pathways of plants to salt stress include changes in Ca^2+^ levels and accumulation of reactive oxygen species (ROS). After perceiving salt stress, plant cells activate calcium signaling and form complexes to maintain Na^+^/K^+^ balance within the cells [6]. Additionally, ROS also plays a regulatory role in plant growth and development, and the plants produce antioxidants to remove excess ROS and maintain homeostasis [7]. Plant hormones play a crucial role in responding to salt stress, which could be divided into ABA-dependent and ABA-independent types [8]. During salt stress, ABA can be rapidly synthesized and released, which activates the kinase cascades while also regulating water and osmotic homeostasis in plants. Several transcription factors, also known as cis-acting factors, have been reported to be associated with response to salt stress, which include MYB, WRKY, bHLH, bZIP and NAC [9]. Additionally, plants also respond to salt stress by regulating osmotic homeostasis [10]. For example, they increase the production of substances such as proline to regulate plant osmotic pressure [11]. Although there have been many reports on the mechanisms of plant response to salt stress, they are still not comprehensive enough in mangroves.

Mangroves grow in swamps and coastal areas where sea waters and fresh waters meet in tropical and subtropical regions [12]. They play important ecological roles, such as acting as windbreaks, and in wave attenuation, land building, providing habitats for birds and purifying the ocean. Mangroves have attracted attention from some experts as high-quality natural salt-tolerant breeding materials. As early as 2002, Banza et al. [13] used transcriptome sequencing technology to identify differentially expressed genes (DEGs) in response to salt stress in *Bruguiera gymnorhiza*. In the same year, Yamada et al. [14] cloned *allene oxide cyclase* (*AOC*) from *Bruguiera sexangula* and demonstrated that this gene can enhance salt tolerance in tobacco (*Nicotiana tabacum)*. Subsequently, several researchers have isolated other genes in mangroves that respond to salt stress, such as *catalases* (*CATs*), *ascorbate peroxidases* (*APXs*), *peroxidases* (*POXs*), *glutathione reductases* (*GRs*) and *plasma membrane intrinsic proteins* (*PIPs*) [15]. Although there have been many reports on the regulatory mechanism of salt stress in mangroves, they are still not comprehensive and systematic. *Sonneratia apetala* Buch.-Ham., also known as mangrove, is a salt-secreting tree that belongs to the *Sonneratiaceae* family [16]. It is native to Bangladesh and India and is an important protected plant in China. Currently, there are relatively few reports on the molecular response mechanisms of *S. apetala* against salt stress. It is necessary to conduct in-depth research on the molecular response mechanisms to salt stress in *S. apetala*, which we have addressed here.

In recent years, a rapid development in high-throughput sequencing technologies has expedited the studies on plant salt stress response. Third-generation sequencing technologies have advantages, such as fast read speed, long read lengths and high accuracy. Transcriptome sequencing technologies that combine single-molecule real-time sequencing (SMRT) and RNA sequencing have played a crucial role in deepening our understanding of plant salt tolerance mechanisms, and they have been widely applied in plants such as *Arabidopsis*, cotton (*Gossypium* spp.) and *Casuarina equisetifolia* [6]. Such technologies could provide important technical support for transcriptome analysis of *S. apetala*, whose whole genome sequence has not been reported. In this study, we combined SMRT and RNA sequencing technologies to analyze the transcriptome of *S. apetala*. Differentially expressed transcription factors in *S. apetala*, under salt stress, were analyzed along with the exploring of regulatory mechanisms of signal transduction, ion homeostasis and ROS homeostasis. This study provides a theoretical basis for revealing the mechanisms of salt stress response of *S. apetala* and for salt-tolerance breeding.

## 2. Results

### 2.1. Identification of Growth and Physiological Indexes after Salt Stress

**Figure 1:**
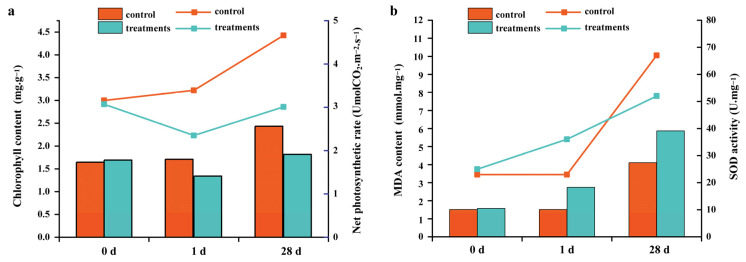
Effects of salt stress on photosynthetic characteristics and membrane protection system of *S. apetala*. (**a**) Effects of salt stress on chlorophyll content and net photosynthetic rate. (**b**) Effects of salt stress on MDA content and SOD activity. MDA, malondialdehyde; SOD, superoxide dismutase. Histogram represented chlorophyll content (**a**) and MDA content (**b**); line chart represented net photosynthetic rate (**a**) and SOD activity (**b**).

The growth indices differed significantly between the control and salt-treated groups of *S. apetala* (Table 1). Compared with the control group, the increments in root length, stem growth, leaf number, leaf length and plant fresh mass were significantly reduced in the salt stress treatment group. Also, the net photosynthetic rates and chlorophyll content of plants decreased on day 1 and day 28 of salt treatment (Figure 1). Further, malondialdehyde (MDA) content and the activity of superoxide dismutase (SOD) were determined at different growth stages. MDA content increased at different stages of salt stress treatment as compared with normal culture conditions, indicating that membrane lipid peroxidation damage occurred in the leaf tissues of *S. apetala* under salt stress. SOD is the primary contributor of scavenging free radicals in plants, and its activity directly affects the content of MDA [17]. In this study, SOD activity and MDA content showed opposite trends under long-term salt stress (28 d). SOD activity was significantly higher than in the control group at 1 day after salt stress treatment, but it was lower than that of the normal culture condition at 28 days. This could be because the metabolic balance of reactive oxygen species was broken in the long-term high-salinity environment, and the activity of free radicals was enhanced, which leads to oxidative damage to the membrane structure and damage to the membrane protection system in the body of *S. apetala*. These results indicate that the growth and physiological changes were obvious at different stages of salt stress treatment, and some salt-sensitive genes may be involved in the regulation of this process.

### 2.2. An Overview of Analysis of Sequencing Data

The study utilized the PacBio sequencing platform to sequence a single library, which generated a total of 295,501 full-length transcripts, with an average length of 1418 bp. To explore the gene expression pattern of *S. apetala* under salt stress, we generated a total of nine cDNA libraries for three types of samples: a control (0 d) and two treatment groups (1 d and 28 d), each with three biological replicates. A total of 3.95 Gb raw reads were generated, with an average of approximately 438,000 reads per sample (Table 2). After filtering, 388.45 Mb of clean reads were obtained, with over 90% of the bases having mass values ≥ 20 (Q20) and 30 (Q30). For all the samples, bases with a mass value of 37 represented the highest percentage (Figure S1). Filtered reads were screed to the *S. apetala* full-length transcriptome sequence, and the mapping rate for each library ranged from 88% to 90.11% (Table 2). Further, the correlation between replicates was high, as determined by the Pearson correlation coefficient (Figure S2). These results indicate that the sequencing data were reliable.

### 2.3. Analysis of Differentially Expressed Genes (DEGs) and Co-DEGs Cluster

**Figure 2:**
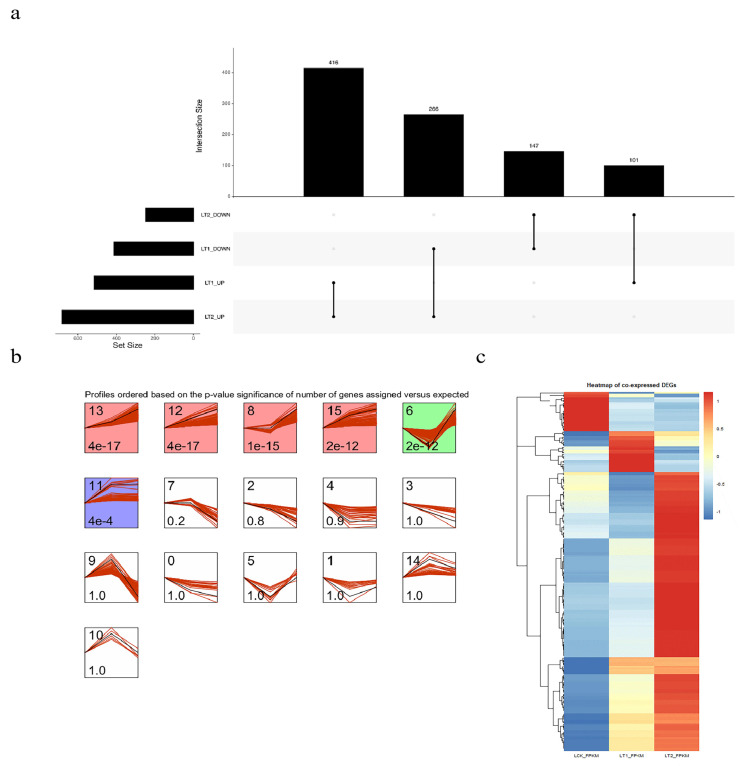
Differentially expressed *S. apetala* genes in response to salt stress. (**a**) Venn diagram shows the results of overlap after the analysis of differential expression of genes (|log_2_FC| ≥ 1) at two time points. (**b**) Analysis of time sequence patterns of co-expressed DEGs. Different background colors represented different clusters. Among them, the white background (profiles 2, 3, 4, 5, 7, 9, 10, 14) represented the gene set with insignificant clustering, the red background (profiles 8, 12, 13, 15) represented the gene set with up-regulated expression with salt stress time, the green background (profile 6) represented the gene set with down-regulated expression in LT1 and up-regulated expression in LT2 and the purple background (profile 11) represented the gene set with unchanged expression after LT1 up-regulation. (**c**) Transcript abundance of the co-expressed DEGs in control (LCK), 1 day (LT1) and 28 days (LT2) salinity treatment of *S. apetala*.

**Figure 3:**
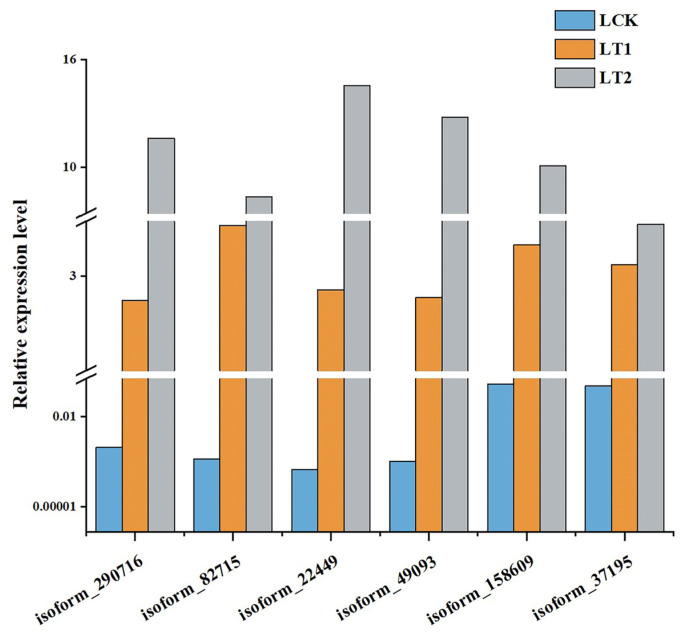
Relative transcript levels of the six randomly selected genes with co-expressed patterns through quantitative real-time polymerase chain reaction (qRT-PCR) in control (LCK), 1 day (LT1) and 28 days (LT2) salinity treatment of *S. apetala*.

To compare the gene expression levels and identify DEGs, the statistic of number of fragments per kilobase of transcript per million mapped reads (FPKM) was utilized. A total of 4712 DEGs (|log_2_FC| ≥ 1, *p*-value < 0.01, Q-value < 0.05) were identified during the salt stress response of *S. apetala*. Among them, 1653 were up-regulated and 1500 were down-regulated in LT1 (1 d), while 1645 were up-regulated and 844 were down-regulated in LT2 (28 d). As indicated in the Venn diagram, 930 genes were expressed differentially at both the time points and were defined as co-expressed DEGs. So, we hypothesized that these (930) genes might play a key role in the response to salt stress in *S. apetala* (Figure 2a). Short-Term Sequence Expression Miner (STEM), a Java-based software, is the first program to analyze short-term sequence microarray gene expression [18,19]. In order to understand the expression profile of co-expressed DEGs, the STEM software (v1.1) was used and six significant profiles (*p*< 0.05) from three clusters were identified (Figure 2b,c). We randomly selected six co-expressed DEGs and examined their transcript levels via quantitative real-time PCR (qRT-PCR) and confirmed a concordance with the expression values from high-throughput sequencing data (Figure 3).

### 2.4. Functional Categorization of Deferentially Expressed Genes

**Figure 4:**
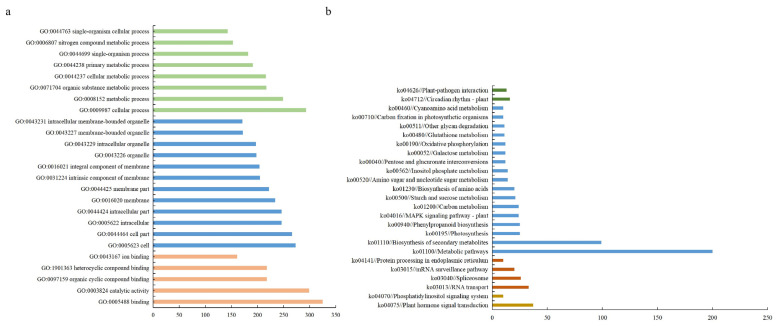
GO (**a**) and KEGG (**b**) classification of the co-expressed DEGs under different salt stress conditions.

Co-expressed DEGs serve as a reflection of the biological response of organisms to varying conditions [20]. Studying co-expressed DEGs contributes to the identification of novel functional genes, construction of biological pathways and establishment of regulatory networks for transcription factors. To investigate the mechanism of response for *S. apetala* leaves to salt stress, a Gene Ontology (GO) analysis was conducted on 930 co-expressed DEGs. A total of 715 DEGs were successfully annotated in the GO database (Figure 4a). The significantly enriched subcategories in biological processes were mainly related to biological metabolism (26.8%) and cellular processes (31.5%). In terms of cellular components, the subcategories mainly related to cellular components (28.6%) and cells (29.4%) were significantly enriched. In terms of molecular functions, the significantly enriched subcategories were mainly related to catalytic activity (32.2%) and binding (34.9%).

Further, 444 co-expressed DEGs were successfully annotated in the KEGG enrichment analysis (Figure 4b). Here, the co-expressed DEGs were enriched in five major pathways, including metabolism (49.6%), genetic information processing (27.5%), environmental information processing (12.5%), organismal systems (6.0%) and cellular processes (4.4%). The most enriched pathways were the metabolic pathway (ko01100), RNA transport (ko03013), plant hormone signal transduction (ko04075) and the plant circadian rhythm pathway (ko04712), respectively. In addition, several pathways related to salt stress, such as the plant MAPK signaling pathway (ko04016), oxidative phosphorylation (ko00190) and phenylpropanoid biosynthesis (ko00940), were also significantly enriched. These results implied the vital role of the common differentially expressed genes in the response to salt stress in the leaves of *S. apetala*.

#### 2.4.1. Annotation of Transcription Factors in Co-Expressed DEGS

**Figure 5:**
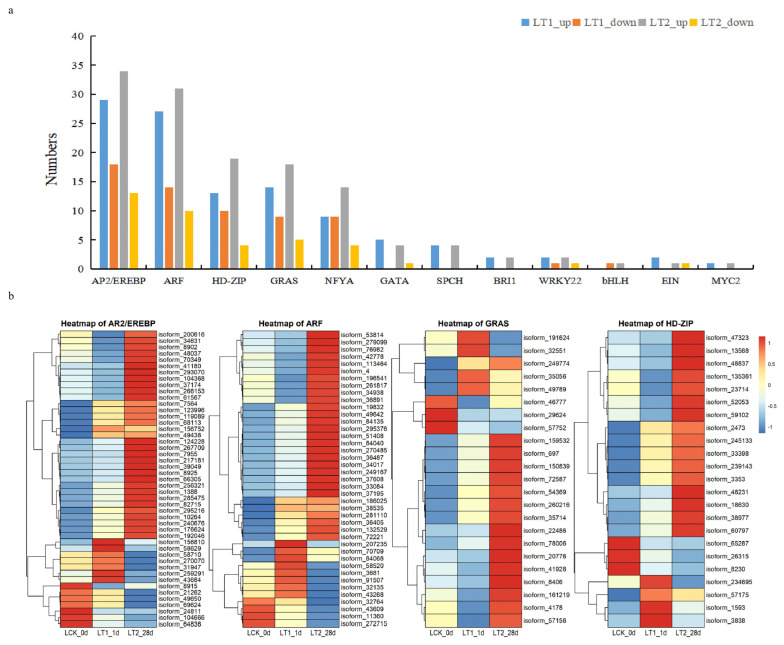
Differentially expressed transcription factors under salt stress. (**a**) Statistics of co-differential expression of transcription factors. (**b**) Heatmap of the four transcription factor families that harbored maximum number of members/genes.

In this study, many transcription factors (TFs) are affected by salinity conditions; a total of 170 co-expressed DEGs were identified as TFs (Figure 5; Supplementary Data S1). The top five families were AP2/EREBP, ARF, HD-ZIP, GRAS and NF-YA, and corresponded to 47, 43, 23, 23 and 18 genes in each, respectively. We studied the expression of these transcription factors in the control and treatment groups (Figure 5; Supplementary Data S1). We found that 108 TFs were up-regulated and 62 were down-regulated in LT1, whereas, in LT2, 131 TFs were up-regulated and 39 were down-regulated. In general, most of the TFs maintained high expression in LT2. In addition, more than half of the common differentially expressed transcription factor genes showed a continuous upward trend across LT1 and LT2, indicating that they might play a crucial role in the response of *S. apetala* leaves to salt stress.

#### 2.4.2. Co-Expressed DEGs Involved in Signal Transduction under Salt Stress

**Figure 6:**
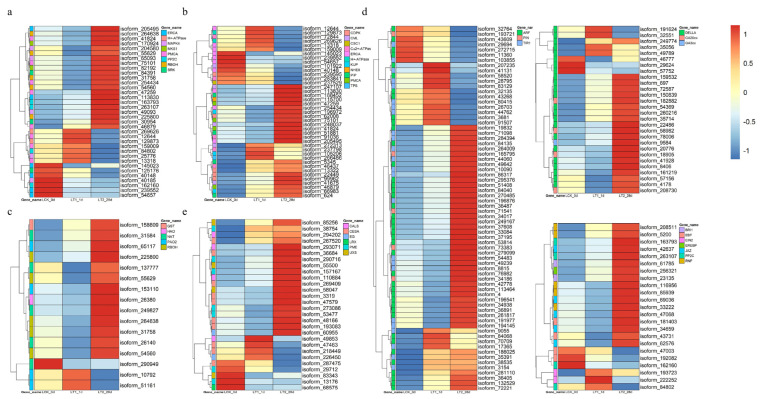
Heatmap of co-expressed DEGs related to signal transduction (**a**), ion homeostasis and water transport (**b**), reactive oxygen species production and scavenging (**c**), hormone response (**d**) and cell wall modification (**e**).

In this study, we found that there were at least five co-expressed DEGs related to the production of ROS (*respiratory burst oxidase homolog protein C-like*, *RBOH*) (Figure 6a; Supplementary Data S2) in both LT1 and LT2. These five genes were all up-regulated in LT2, while only one gene was up-regulated in LT1. Additionally, this study identified four co-expressed DEGs of the *mitogen-activated protein kinase 4* (*MAPK4*) family, among which one was up-regulated in both the LT1 and LT2 groups. Other co-expressed DEGs involved in salt stress signal transduction include *plasma membrane calcium-transporting ATPase* (*PMCA*), *endoplasmic reticulum calcium-transporting ATPase* (*ERAC*), *calcium-dependent protein kinase* (*CDPK*), *calcium-binding protein CML* (*CML*), which related to calcium signal transduction, and protein phosphatase 2C (PP2C), which related to plant hormone signal transduction.

#### 2.4.3. Co-Expressed DEGs Involved in Ionic Balance and Water Transport

The disruption of ion homeostasis is considered as a primary factor for the restricted growth of plants experiencing salt stress. An excessive buildup of sodium ions could alter the levels of calcium, thereby upsetting the equilibrium of intracellular ion concentrations [21]. We observed twenty-nine co-expressed DEGs related to ionic balance and four related to water transport (Figure 6b; Supplementary Data S3). The significance of Ca^2+^ as a secondary messenger is widely recognized [22]. We discovered that 25 co-expressed DEGs were associated with Ca^2+^-signaling. Among them, *calcium-dependent protein kinases* (*CDPK*; three genes), *probable calcium-binding protein CML36* (*CML*; three genes), *plasma membrane-type-like Ca^2+^-transporting ATPase* (*PMCA*; two genes) and *endoplasmic reticulum-type-like Ca^2+^-transporting ATPase* (*ERAC*; one gene) were simultaneously up-regulated under salt stress. Three DEGs encoding potassium uptake protein (KUP) were also detected under salt stress. These genes are reported as potassium transporters. Furthermore, we found a sodium/hydrogen exchanger gene (*NHE8*) and three copper-transporting ATPase RAN1 genes (*Cu^2+^-ATPase)* showing differential expressions. Six water channel protein genes (*PIPs*) exhibited increased accumulation at both of the two time points of *S. apetala* salt stress.

#### 2.4.4. Co-Expressed DEGs Involved in Production and Scavenging of Reactive Oxygen Species

To investigate a possible role of ROS-related genes in the salt response of *S. apetala*, we analyzed the accumulation characteristics of salt-responsive genes associated with ROS. In total, 34 co-expressed DEGs, including 14 up- and 20 down-regulated genes in LT1, whereas 24 up- and 10 down-regulated in LT2 were associated with ROS signaling (Figure 6c; Supplementary Data S4). In the LT1 group, we observed that *respiratory burst oxidase* (*RBOH*) and cytochrome P450 family genes associated with the production of ROS were up-regulated. In LT2, three genes, encoding for *glutathione S-transferase* (*GST*), *polyamine oxidase* (*PAO*) and *nucleobase-ascorbate transporter* (*NAT*), involved in ROS scavenging, continued to be up-regulated.

#### 2.4.5. Co-Expressed DEGs Involved in Hormone Response

Plant hormones play a vital role in regulating growth, development and molecular signal transduction in response to salt stress. We found that ‘plant hormone signal transduction’ was an enriched pathway in co-expressed DEGs of *S. apetala* treated with salt stress. We found 162 co-expressed DEGs involved in hormone response, of which 98 were up- and 64 were down-regulated in LT1, while 122 were up- and 40 were down-regulated in LT2, respectively (Figure 6d; Supplementary Data S5). We identified co-expressed DEGs associated with various plant hormones, including auxin, ethylene, brassinosteroids, abscisic acid and gibberellin. Specifically, we observed changes in the expression levels of genes encoding TFs or functional enzymes, such as auxin response factor (ARF), transport inhibitor response 1 (TIR1), probable auxin efflux carrier component 1b (related to auxin signaling), EIN3-binding F-box protein, ethylene-insensitive protein 2 (EIN2), EREBP-like factor (associated with ethylene signaling), protein brassinosteroid insensitive 1 (BRI1, related to brassinosteroid signaling), RING-H2 finger protein, RNF (associated with abscisic acid signaling) as well as ethylene signaling-relating proteins gibberellin 3beta-dioxygenase (GA3ox), gibberellin 20-oxidase (GA20ox) and DELLA proteins. Differential expressions of such genes indicates that these hormone signaling pathways may play a role in a plant’s response to salt stress.

#### 2.4.6. Co-Expressed DEGs Involved in Cell Wall Synthesis or Modification

In this study, a total of 28 co-expressed DEGs were related to cellulose synthesis, pectin catabolism and callose synthesis (Figure 6e; Supplementary Data S6). These genes include *callose synthase* (*CALS*, four genes), *cellulose synthase A* (*CESA*, ten genes), *endoglucanase* (two genes), *pectinesterase* (seven genes) and *UDP-glucuronate decarboxylase* (*EG*, five genes). Specifically, all the callose synthase genes were up-regulated in LT1, while only three of them were up-regulated in LT2. Three *cellulose synthase A* (*CESA*) genes, three *pectinesterases* (*PME*) and two *UDP-glucuronate decarboxylase* (*UXS*) genes were continuously up-regulated at both time points (LT1 and LT2). Additionally, an *endoglucanase* (*EG*) gene was up-regulated in both LT1 and LT2. These findings indicate that these genes may play important roles in response to salt treatment and might involve genes related to cellulose synthesis, pectin catabolism and callose synthesis.

## 3. Discussion

This study elucidates the salt stress response mechanism of *S. apetala*. Salt stress can cause various types of damage to plants, which could ultimately result in their death. Investigating the mechanism and enhancing salt tolerance in plants has been a prominent focus of research for numerous years. Currently, this research is primarily concentrated on model plants. A lack of published genomes for non-model plants has posed a challenge for molecular research in such species. In this scenario, Iso-seq (isoform sequencing) offers crucial technical support and several advantages, including high throughput and an ability to generate lengthy fragments. Despite being a halophyte, with a high salt tolerance capacity, only a limited number of mechanistic studies have been conducted on *S. apetala* [23]. Our study provides a first draft of the leaf transcriptome of this mangrove plant (*S. apetala*) by analyzing the salt stress response at two time points with the help of a combination of PacBio long-read RNA sequencing and RNA-seq sequencing methods. The differential gene expression at each time point was determined at the two treatment time points (1 day and 28 days) when the transcriptomes were compared to a control group (0 days). A large number of DEGs were identified at both the time points. To explore the common expression patterns of genes under salt stress in *S. apetala* leaves and to further investigate the gene regulatory mechanism and gene function, the STEM software was used, which generated fifteen profiles and four clusters. Particular attention was paid to the pathways related to salt stress signal transduction, hormone response, ion homeostasis, reactive oxygen species scavenging and cell wall synthesis or modification.

The role of transcription factors, such as MYB, MYC, WRKY and AP2/EREBP, has been well documented during response to salt stress [17]. In this study, 170 TFs were found among the co-DEGs (Figure 5; Supplementary Data S1), where the most abundant family was AP2/EREBP, followed by ARF, HD-ZIP and GRAS. AP2/EREBP is one of the three major gene families in *Arabidopsis* [24], and it plays a crucial role in regulating a plant’s response and adaptation to various abiotic stresses, including salinity, drought and high temperature [25]. Auxin response factors (ARFs) are important transcription factors involved in the auxin signaling pathway. The TIR1/AFB-Aux/IAA-ARF signaling pathway is one of the most important signaling pathways in plants [26]. In this study, a large number of *ARF* genes were up-regulated, indicating a possible role of auxin signaling. Additionally, 11 *TIR1* genes were simultaneously up-regulated in the LT1 and LT2 treatment groups. We inferred that the TIR1/AFB-Aux/IAA-ARF signaling pathway might integrate salt stress signals into the auxin-related gene regulatory network to respond to salt stress in *S. apetala*. In this study, we also identified many transcription factors related to plant hormones that were differentially co-expressed in the LT1 and LT2 treatment groups, such as the WRKY, BRI1, EIN, MYC and GRAS families. Two *WRKYs* and one *MYC* were found to be consistently up-regulated under salt stress, implying their potential roles in the salt stress response of *S. apetala*, which is consistent with previous reports on the transcription factors involved in plant responses to salt stress [27].

Previous research indicates that plants sense salt stress signals through unidentified receptors and subsequently transmit them through a network of signaling pathways involving aspects such as ion homeostasis, osmotic protection and ROS [28]. In this study, it was evident that the signal transduction during salt stress response in *S. apetala* also encompasses these pathways (Figure 6a; Supplementary Data S2). Ion stress signaling pathways mainly rely on ions such as H^+^ and Ca^2+^. Calcium ions, as a second messenger in plants, play a crucial role during response to salt stress [29]. The Ca^2+^ signaling pathway mainly consists of three steps: Ca^2+^ signal generation, perception and recognition and signal transduction. Previous studies have found that calmodulins (CaMs), calmodulin-like proteins (CMLs) and calcineurin B-like proteins (CBLs) are involved in Ca^2+^ signal perception. Ca^2+^-dependent protein kinases (CDPKs/CPKs) and Ca^2+^/CaM-dependent protein kinases (CCaMKs) are components of Ca^2+^ signal response [30]. Under salt stress, the accumulation of Ca^2+^ in plant cells is perceived by SOS3 and regulates the transcription level of genes. Bacha et al. [31] demonstrated that the increase in Ca^2+^ in plant cells could regulate the expression of *LeHAK5* and improve the salt tolerance of tomato (*Solanum lycopersycum*). As a Ca^2+^ signal sensor replay, the *CML* genes have been reported to be involved in abiotic stress signaling [32]. Du et al. [33] found that the *MsCML46* from *Medicago sativa* promoted signal transduction by binding free Ca^2+^ and maintained a high K/Na ratio, which ultimately improved the salt tolerance of tobacco. Moreover, CDPK, as the Ca^2+^ signal sensor responder, plays a vital role in response to drought stress in *Gossypium barbadense* [32]. In this study, a total of five *CMLs* and a few *CDPKs* were identified in the set of co-expressed DEGs. Specifically, in LT1, three *CDPK* and three *CML* genes were up-regulated, while, in LT2, four *CDPK* and four *CML* genes were up-regulated. This indicates that, in an early stage of salt stress, *S. apetala* responds by up-regulating both the Ca^2+^ sensing and the signal response components. Under prolonged salt stress, the *CML* and *CDPK* genes maintain a high level of expression, which might promote further response of the organism to the saline environment. *CML* and *CDPK* genes are also involved in the regulation of the salt overly sensitive (SOS) pathway in plants during their response to salt stress [34]. *CML* and *CDPK* genes can interact with key proteins in the SOS pathway and could regulate the activity and function of these proteins [35]. For example, in the SOS pathway, CBL could activate CIPK in response to salt stress and thus could promote ion excretion. CML and CDPK, on the other hand, can interact with CBL and CIPK, which could regulate their activity and stability [36]. Therefore, we infer that the SOS pathway is also likely to play an important role in signal transduction during salt stress response in *S. apetala*. Aside from CML and CDPK, Ca^2+^-transporters, such as Ca^2+^-ATPase and calcium-permeable stress-gated cation channel (CSC1), not only participate in Ca^2+^-dependent signal transduction pathways but also play a crucial role in maintaining ion homeostasis within cells [37]. Studies have shown that plasma membrane Ca^2+^ transport ATPase (PMCA) mediated the efflux of calcium ions from the cytoplasm under salt stress, thereby maintaining cellular ion homeostasis [38]. CSC1 is a calcium ion channel, and cells overexpressing the *CSC1* gene rapidly accumulated calcium ions to reduce the toxic effects of osmotic stress caused by ions [39]. In our study, two *PMCAs* and one *CSC1* exhibited up- and down-regulated expression at the two times points, respectively (Figure 6a,b; Supplementary Data S2 and S3).

In addition to Ca^2+^, protons (H^+^) are also known as the second messengers in plant stress conditions [40]. The production and transportation of H^+^ can regulate cell pH and potential. Under salt stress, in the plasma membrane, P-type H^+^-ATPase converts ATP into H^+^ and ADP and releases the generated H^+^ to the outside of the cell so that it could regulate the intracellular pH and ion balance while providing energy [19]. Additionally, the mitochondrial inner membrane F-type H^+^-ATPase has a similar function to the P-type H^+^-ATPase. After generating H^+^, the F-type H^+^-ATPase transports it out of the mitochondria to maintain mitochondrial pH and ion balance. In this study, we found one *F-type H^+^-ATPase* and one *plasma membrane H^+^-ATPase* gene were both up-regulated in LT1 and down-regulated in LT2 (Figure 6a; Supplementary Data S2). This indicated that the H^+^-mediated ion signaling pathway could play an important role during the early stages of salt stress.

Also, it is well known that the mitogen-activated protein kinases (MAPKs) play a crucial role in the response to salt stress in plants [41]. The MAPK pathway consists of three types of protein kinases: MAPK, mitogen-activated protein kinase kinase (MAPKK/MAP2K/MKK/MEK) and mitogen-activated protein kinase kinase kinase (MAPKKK/MAP3K/MKKK/MEKK) [42]. In this pathway, MAPKKK, MAPKK and MAPK proteins phosphorylate each other while they transmit signals and regulate the expression of relevant genes in response to stress [19]. The MAPK reaction in plants is highly complex and interacts with multiple signaling pathways. Yoo et al. [43] demonstrated that AtMKK9-AtMAPK6/AtMAPK3 cassette phosphorylates the T174 site of EIN3 in *Arabidopsis* while promoting ethylene signal transduction. In the interaction between MAPK and auxin, Jia et al. [44] found that the AtMKK7-AtMPK6 cascade phosphorylates AtPIN1 while regulating auxin transport. Meanwhile, MAPK can also induct ROS under salt stress. Son et al. [45] found that GmMPK6 induced ROS production through transcriptional regulation of GmRbohI1 and increased salt tolerance in soybean. In our study, 5 *MAPK*4 indicates a possible important role of the MAPK pathway during the salt stress response of *S. apetala* (Figure 6a; Supplementary Data S2).

High concentrations of soluble salts could decrease the osmotic pressure within plant cells, leading to osmotic stress. Osmotic stress signals help plants to reestablish their osmotic balance and play a crucial role in subsequent response to salt stress. Tang et al. [46] demonstrated that the cascading response of osmotic stress signals, mediated by plasma membrane intrinsic proteins (PIPs), plays a significant role in plant salt stress. Kumar et al. [47] demonstrated that overexpression of the *CcPIP1* gene from *Carya cathayensis* enhances the tolerance of *Arabidopsis* to abiotic stress. Vaziriyeganeh et al. [48] found the functional importance of PIP2; 2 in salt tolerance of *Puccinellia tenuiflora*. In our study, five *PIP* genes were identified, among which four were up-regulated in LT1, and all five were up-regulated in LT2 (Figure 6a; Supplementary Data S2). This indicates an important role of *PIP* genes in the signal transduction during salt stress in *S. apetala*.

Sodium–potassium balance is a key factor in maintaining cellular ion homeostasis under salt stress. Previous studies have identified *LrKUP8* [49], a potassium transporter in *Lycium barbarum*, which is up-regulated under high salt stress and confers salt tolerance when transiently expressed in tobacco. In this study, three *KUP* co-expressed DEGs were found under salt stress in *S. apetala*, indicating their involvement in alleviating ion toxicity that could be caused by salt stress. In addition to metal ions, H^+^ also plays an important role in maintaining ion homeostasis under plant salt stress [19]. Sodium–hydrogen exchangers (NHEs) and H^+^-ATPases are important H^+^ transporters in plants [50]. This study identified an *NHE8* gene and two *H^+^-ATPase* genes that were differentially expressed. The *NHE8* gene was continuously up-regulated under salt stress, while *H^+^-ATPase* genes were up-regulated in LT1 and down-regulated in LT2, indicating their different expression patterns under salt stress in *S. apetala* (Figure 6b; Supplementary Data S3). Additionally, the monovalent cation proton antiporter (CPA) superfamily proteins, comprising Na^+^/H^+^ exchanger (NHX), K^+^ efflux antiporter (KEA) and cation/H^+^ exchanger (CHX) family proteins, also play vital functions in the maintenance of sodium–potassium balance [51] and should be focused on in further studies.

Reactive oxygen species (ROS) play a crucial role in various aspects of plant growth and development. On one hand, ROS accumulation serves as a specific molecular regulator for cell signaling and function, leading to a series of morphological, physiological, biochemical and molecular changes in plants during stress conditions [52]. On the other hand, excessive ROS levels could lead to cellular damage [53]. Thus, maintaining ROS homeostasis is critical for ensuring normal plant growth, especially under conditions of salt stress. ROS is generated in plants in response to short-term salt stress to activate Ca^2+^ signaling. Respiratory burst oxidase homologs (RBOHs), also known as NADPH oxidases (NOXs), are involved in plant responses to biotic and abiotic stresses [54]. Soliman et al. [55] overexpressed the *StRBOHA* gene in potatoes (*Solanum tuberosum*), which enhanced its resistance to *Phytophthora infestans*. Xanthine dehydrogenase 1 (XDH1), in leaf mesophyll cells, can remove excess H_2_O_2_, thus protecting the plants from oxidative damage [56]. Nucleobase-ascorbate transporters (NATs) are responsible for transporting xanthine and uric acid in plants [57]. Further, cytochrome P450 monooxygenases (CYPs) are one of the largest gene families in plants that are involved in various biological processes, including biotic and abiotic stress responses. Wang et al. [58] found that *TaCYP81D5* improved the salt tolerance in seedlings and reproductive stages of wheat (*Triticum aestivum*) by accelerating ROS clearance. Glutathione S-transferases (GSTs) are an important target of the plant stress tolerance mechanism. Meng et al. [59] found that *PeGSTU58* was activated by *PebHLH35* and involved in salt tolerance by maintaining ROS homeostasis in *Populus euphratica.* In this study, multiple overlapping co-expressed DEGs involved in ROS balance were identified, including genes encoding respiratory burst oxidases homologs (RBOHs), nucleobase-ascorbate transporters (NATs), glutathione S-transferases (GST) and cytochrome P450s (CYPs) (Figure 6c; Supplementary Data S4). These genes could play similar important roles in maintaining ROS homeostasis during the salt stress response of *S. apetala*.

Plant hormones, including auxins, abscisic acid, gibberellins and jasmonic acid, play a crucial role in plants’ adaptation to their environment. Auxin is involved in regulating plant growth and development, and it plays a positive regulatory role in salt stress response in *Arabidopsis* [60]. In this study, the expressions of *auxin response genes (ARF*) and auxin transport genes (*PIN*) were up-regulated in LT2 compared to LT1 (Figure 6d; Supplementary Data S5). We hypothesize that *S. apetala* may be involved in a growth recovery stage under salt stress at 1 day and 28 days, and auxin promotes the growth of *S. apetala* under salt stress [60]. Next, gibberellins (GAs) are important endogenous hormones that regulate plant nutrition and reproductive growth. GA oxidases (GA20ox, GA3ox, GA2ox) regulate GA homeostasis in plants under salt stress [61]. Overexpression of the *GhGA2ox1* gene in upland cotton (*Gossypium hirsutum*) enhanced its salt and drought tolerance [61]. Zhu et al. [6] conducted transcriptome sequencing on *Sophora alopecuroides* plants exposed to salt stress. The findings revealed an increase in the expression of gibberellic acid (GA) degradation genes. Additionally, the expression of *DELLA* genes, which act as negative regulators of GA signal transduction, was significantly reduced in response to salt stress. In our study, *DELLA* genes were more strongly down-regulated in LT1 compared to LT2. On the other hand, four co-expressed DEGs associated with GA20ox were up-regulated in LT2, while only two were up-regulated in LT1. We predicted that there might be crosstalk between auxin and GA regulation in *S. apetala*, and the corresponding co-expressed DEGs play an important role in its response to salt stress.

Salt stress could limit leaf growth by inhibiting cell elongation and limiting cellulose synthesis. When plants are exposed to salt stress, their cell walls undergo a series of physiological changes, such as an increase in thickness and alterations in integrity [62]. These changes enhance the stability and elasticity of the cell wall, thereby improving a plant’s ability to adapt to salt stress. Moreover, polysaccharides in the cell wall, such as cellulose and pectin, can also serve as signaling molecules that participate in internal signal transduction and regulation in response to salt stress [63]. Shen et al. [62,64] demonstrated that the periderm tissues of the two *Glycyrrhiza* species (*Glycyrrhiza uralensis* and *Glycyrrhiza inflata*) are thickened under salt stress to maintain normal plant metabolism. Yao et al. [62] identified 92 DEGs related to cell wall synthesis or modification in *Lycium barbarum* under salt stress using Illumina high-throughput sequencing. Cellulose synthase (CESA) and callose synthase (Cals) are important enzymes in cell wall and callose synthesis, respectively [65]. Endoglucanase (EG) is an endonuclease that promotes cellulose degradation. Together, all these enzymes have important effects on cell wall fiber synthesis. Studies have shown that single mutants of *Arabidopsis CESA1* and *CESA6* increase salt sensitivity [66]. Feng et al. [65] identified *GhCalSs* in cotton and predicted their involvement in non-biological stress by affecting cellulose elongation rate. In this study, it was found that three *Cals* genes and three *CESA* genes were up-regulated in LT1 and LT2, while an *EG* gene was down-regulated in LT1 and LT2 (Figure 6e; Supplementary Data S6). These results indicated that *S. apetala* may respond to salt stress by increasing cell wall synthesis and reducing water loss. Pectin is an acidic polysaccharide that plays an important role in plant growth, development and stress resistance. Pectinesterase selectively modifies pectin, whose expression affects cell wall hardness and enhances plant salt tolerance. For instance, using gene knockout technology, Yan et al. [67] demonstrated that the pectin methylesterases 31 (PME31) is a positive regulator of salt stress in *Arabidopsis*. In our experiment, three *PME* genes were up-regulated under salt stress, among which *isoform_157167* and *isoform_290716* had log_2_FC values greater than 10 in LT2. We hypothesize that *PME* genes respond to salt stress as positive regulators in *S. apetala*. Next, we looked at the components of hemicellulose, which is a polymer mainly present in the secondary walls. UDP-glucose glucuronate decarboxylase (UXS) is a key enzyme in the formation of hemicellulose [68]. Ni et al. [68] indicate that *OsUXS3* may regulate CAT activity, Na^+^/K^+^ homeostasis and positively regulate salt tolerance in rice by interacting with an *OsCAT*. In this study, four *UXS* genes were found, with two up-regulated in LT1 and three up-regulated in LT2. It is suggested that *UXS* genes might act as positive regulators in the response of *S. apetala* to salt stress, but their specific mechanisms of action require further study.

**Figure 7:**
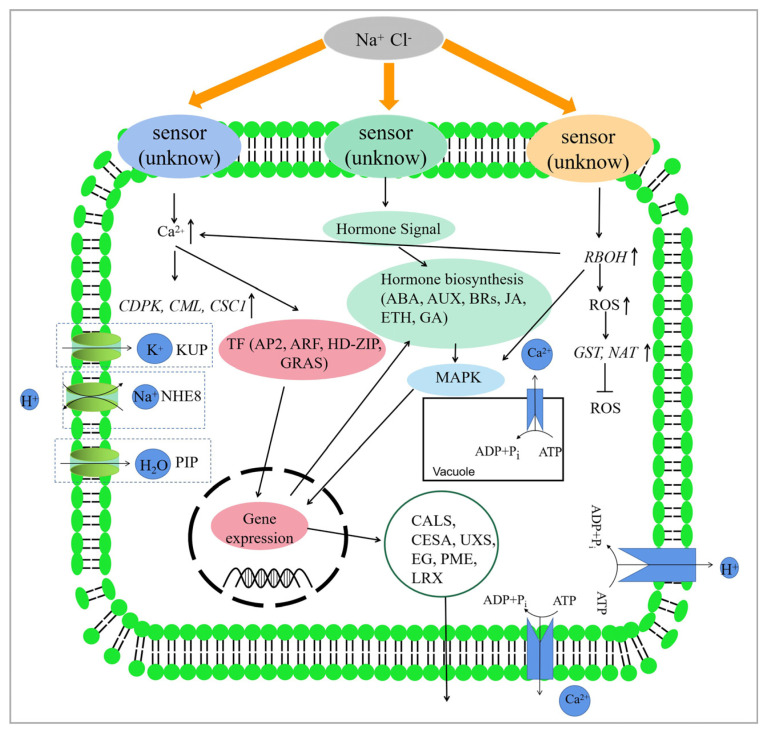
Hypothetical salt stress tolerance mechanism in *S. apetala*.

Based on our findings and previous research, this study proposes a hypothetical molecular mechanism of salt stress response in *S. apetala* (Figure 7). Under salt stress, the receptor located on the plasma membrane perceives the stress signal, which leads to up-regulated expression of the *RBOH* gene and the production of reactive oxygen species (ROS). Calcium ion signals are also activated, and hormone synthesis is regulated to transmit the stress signal. These signals activate downstream genes and trigger salt tolerance mechanisms. *CDPK* and *CML* genes in the Ca^2+^ signaling pathway are activated to transmit the salt stress signal downstream and interact with the MAPK pathway. This would regulate the expression of transcription factors. *NHE8* and *Ca*^2+^-*ATPase* genes are up-regulated to remove excess Na^+^ and Ca^2+^ from the cytoplasm or organelles while maintaining ion homeostasis and H^+^ balance. ROS production activates downstream signals, leading to differential expression of *GST* genes to maintain ROS homeostasis in the cell. Additionally, genes involved in cell wall synthesis or modification are regulated, resulting in cell wall thickening or extension to reduce water loss. Finally, genes involved in intracellular proteins in the plasma membrane are up-regulated to accelerate water uptake and reduce the harmful effects of osmotic stress in a saline environment.

## 4. Materials and Methods

### 4.1. Experimental Materials

Seeds of mature *S. apetala* plants, growing in the coastal mangrove zone of Techeng Island, Guangdong, China (21°09′~21°10′ N, 110°25′~110°27′ E), were collected and buried in artificial soil on a seedbed. After approximately 70 days, seedlings reaching a 10–16 cm height were transplanted into polyethylene bags and watered every other day. After one year of growth, uniformly developed seedlings were selected and divided into experimental groups of control (LCK_0 d) and treatments (LT1_1 d, LT2_28 d). Treatment group samples grown on sandy soil were watered with 300 mmol/L NaCl for 1 day and 28 days, respectively; the control group received the same amount of water. Three biological replicates were included for each of the control and treatment groups (a total of nine plants). The leaves of the seedlings were harvested and immediately placed in liquid nitrogen before storing them at −80 °C for later use.

### 4.2. RNA Preparation, Library Construction and Sequencing

Total RNA was extracted from leaves of nine samples of *S. apetala* with the help of a RNeasy Plant mini kit (Qiagen, Hilden, Germany) according to the manufacturer’s protocol. The RNA quality and integrity were determined by a NanoDrop 2000 (Thermo Scientific, Waltham, MA, USA) and an Agilent 2100 Bioanalyzer (Agilent Technologies, Santa Clara, CA, USA) with OD260/280 values between 1.8 and 2.2 and RIN (RNA integrity number) > 7.0. Qualified RNA was stored at −80 °C for further PacBio and BGISEQ library construction, respectively.

To identify as many transcripts as possible, we combined equal amounts of total RNA from the nine replicates, as well as total RNA extracted from root tissues of *S. apetala* after salt treatment for 0, 7 days (200 and 400 mmol/L NaCl), and 14 days (300 mmol/L NaCl) for the construction of PacBio sequencing library. The mixed RNA sample was reverse-transcribed into cDNA with the help of a SMARTer^TM^ PCR cDNA Synthesis Kit, and the second-strand cDNA was synthesized by PCR. The double-stranded cDNA was then subjected to secondary PCR amplification. The amplified double-stranded DNA was repaired for damage, end-repaired and ligated with SMRT adapters before constructing the full-length transcriptome sequencing library. Then, the SMRT library was sequenced to a data size of 30 GB through the PacBio platform following the methods described by Chen et al. [23].

### 4.3. RNA-Seq and Differentially Expressed Genes Identification

RNA-seq library construction and sequencing to 6 GB size of data was carried out according to the methods described by Li et al. [69]. RSEM [70] was used to determine the short-read data (length of 150 bp) and using the resulting full-length transcripts as a reference sequence. The counts of transcript isoforms were converted into fragments per kilobase of transcript per million mapped reads (FPKM) values, with the help of following formula: FPKM = (transcript reads) × 10^9^/(transcript length × total mapped reads in run). Differential gene expression was determined on the basis of the criteria of |log_2_(Fold change)| ≥ 1, *p*-value < 0.01 and Q-value < 0.05, and the DEseq2 [18,71] software was utilized for an inter-group differential analysis. The differentially expressed genes overlapping in both LT1 and LT2 were defined as co-expressed DEGs for subsequent analysis. The library construction and sequencing were carried out by Beijing Genomics Institute (BGI), China.

### 4.4. GO and KEGG Enrichment Analysis of Co-Expressed DEGs

The 930 identified co-expressed DEGs were functionally classified on the basis of their annotations from GO (Gene Ontology) and KEGG (Kyoto Encyclopedia of Genes and Genomes) databases. Additionally, enrichment analysis was performed with the help of the phyper function in the R software (v4.2.3).

### 4.5. Quantification of Transcript Levels via Quantitative Real-Time PCR (qRT-PCR)

cDNAs were obtained by reverse transcription of total RNAs from nine leaf samples of *S. apetala* (LCK_a, LCK_b, LCK_c, LT1_a, LT1_b, LT1_c, LT2_a, LT2_b and LT2_c). qRT-PCRs were conducted on a CFX Connect™ Real-Time PCR Detection System through SYBR green-based real-time PCR. The Light-Cycler FastStart DNA Master SYBR Green (Roche Applied Science, Mannheim, Germany) was adopted with gene-specific primers designed through Primer Premier v5 software (Table S1). The PCR samples were incubated at 94 °C for 5 min and 40 cycles of 30 s at 94 °C, 30 s at 57 °C and 72 °C for 30 s. The 2^−△△Ct^ method was used to calculate the abundance of each gene against 18S RNA of *S. apetala* (GenBank number KJ161168). All reactions were run in triplicate to ensure reproducibility and reliability.

## 5. Conclusions

Using ISO-seq and RNA-seq technologies, we have studied the molecular response of *S. apetala* under salt stress. A total of 4712 DEGs were identified in this study, with 930 of them being co-differentially expressed at two time points. After annotations, key genes involved in the salt stress response of *S. apetala* were selected. These genes included a large number of hormone response and signal transduction genes, which may play important roles in the salt stress response of *S. apetala* leaves. In addition, this study also discovered differentially expressed genes related to ion homeostasis (*CDPK*, *CML*, *KUP* and *NHE*), water transport (*PIP*), reactive oxygen species homeostasis (*RBOH* and *GST*) and cell wall synthesis and modification (*CELA*, *Cals*, *PME*, *EG*, *LRX* and *UXS*). All these genes are expected to play important roles in the salt stress response of *S. apetala*. Also, these results suggest that the response of *S. apetala* leaves to salt stress involves a network system in which multiple genes and pathways are co-regulated.

In summary, this study offers valuable insights into the intricate molecular mechanisms underlying the response of *S. apetala* to salt stress and provides a foundation for future efforts to enhance salt stress tolerance.

## Figures and Tables

**Table 1:** Growth changes in the control and treatment groups after salt stress for 28 days.

Sample	Increment of Root (cm)	Increment of Stem Length (cm)	Increment of Stem Base Diameter (cm)	Increment of Leaf Number (Piece)	Increment of Leaf Length (cm)	Increment of Leaf Width (cm)	Increment of Seedlings Fresh Weight (g)
Control	8.01 ± 0.22 ^a^	1.42 ± 0.16 ^a^	0.45 ± 0.01 ^a^	2.43 ± 0.04 ^a^	0.68 ± 0.04 ^a^	0.18 ± 0.02 ^a^	0.26 ± 0.05 ^a^
Salinity treatments	7.33 ± 0.30 ^b^	1.13 ± 0.02 ^b^	0.37 ± 0.08 ^ab^	2.06 ± 0.11 ^b^	0.51 ± 0.03 ^b^	0.15 ± 0.06 ^ab^	0.18 ± 0.03 ^b^

Note: Significant differences occurred between letters “a” and “b” (*p* < 0.05).

**Table 2:** Statistics of sequencing data in all the samples.

Sample	Total Raw Reads (M)	Total Clean Reads (M)	Total Clean Bases (Gb)	Clean Reads Q20 (%)	Clean Reads Q30 (%)	Clean Reads Ratio (%)	Total Mapping (%)
LCK_a	43.78	43.16	6.47	97.13	92.33	98.58	89.77
LCK_b	43.78	43.27	6.49	97.21	92.65	98.84	89.37
LCK_c	43.78	43.21	6.48	97.26	92.38	98.7	90.11
LT1_a	43.78	43.02	6.46	96.86	92.31	98.26	88.43
LT1_b	43.78	43.15	6.47	97.29	92.46	98.56	88
LT1_c	43.78	43.26	6.49	97.07	92.29	98.81	88.37
LT2_a	43.78	43.11	6.47	97.23	92.46	98.47	88.96
LT2_b	43.78	43.06	6.46	97.07	92.39	98.36	88.62
LT2_c	43.82	43.21	6.48	96.96	92.16	98.61	89.07
Sum	394.06	388.45	58.27				

Note: Q20, percentage of bases with a phred value of at least 20; Q30, percentage of bases with a phred value of at least 30.

## Data Availability

The sequence data reported in this paper have been deposited in the Genome Sequence Archive (https://ngdc.cncb.ac.cn/gsa/, accessed on 10 November 2023), Chinese Academy of Sciences [72]. Sequence data are available under accession numbers CRA006866 (https://ngdc.cncb.ac.cn/gsa/s/ZfnE3R4y, accessed on 10 November 2023) and CRA006863 (https://ngdc.cncb.ac.cn/gsa/s/1W0K5srS, accessed on 10 November 2023).

## Supplementary Materials

The following supporting information can be downloaded at: https://www.mdpi.com/article/10.3390/plants12223849/s1, Figure S1: Base quality analysis of clean reads for sequencing library of LCK_a (a), LCK_b (b), LCK_c (c), LT1_a (d), LT1_b (e), LT1_c (f), LT2_a (g), LT2_b (h) and LT2_c (i).; Figure S2: Pearson correlation analysis between samples based on gene expression profiles; Table S1: The primer sequences used for quantitative real-time PCR (qRT-PCR) analysis.
