# Supplementary material for: PacBio Full-Length Transcriptome Sequencing Reveals the Mechanism of Salt Stress Response in Sonneratia apetala
Source: Plants (Basel). 2023 Nov 14;12(22):3849. doi: 10.3390/plants12223849 (PMC10675792; doi:10.3390/plants12223849)
Supplement: Supplementary file 1 [file plants-12-03849-s001.zip › Figures and Supplementary Files/Supplementary Figures and Table.pdf]

Supplementary Material

The following Supporting Information is available for this article:

Supplementary Figure S1. Base quality analysis of clean reads for sequencing library of LCK_a (a), LCK_b (b), LCK_c (c), LT1_a (d), LT1_b (e), LT1_c (f), LT2_a (g), LT2_b (h) and LT2_c (i). LCK, control; LT1, salinity treatments for 1 d; LT2, salinity treatments for 28 d. a, b, c represent the three repetitions.

Supplementary Figure S2. Pearson correlation analysis between samples based on gene expression profiles. LCK, control; LT1, salinity treatments for 1 d; LT2, salinity treatments for 28 d. a, b, c represent the three repetitions.

Supplementary Table S1. The primer sequences used for quantitative real-time PCR (qRT-PCR) analysis.

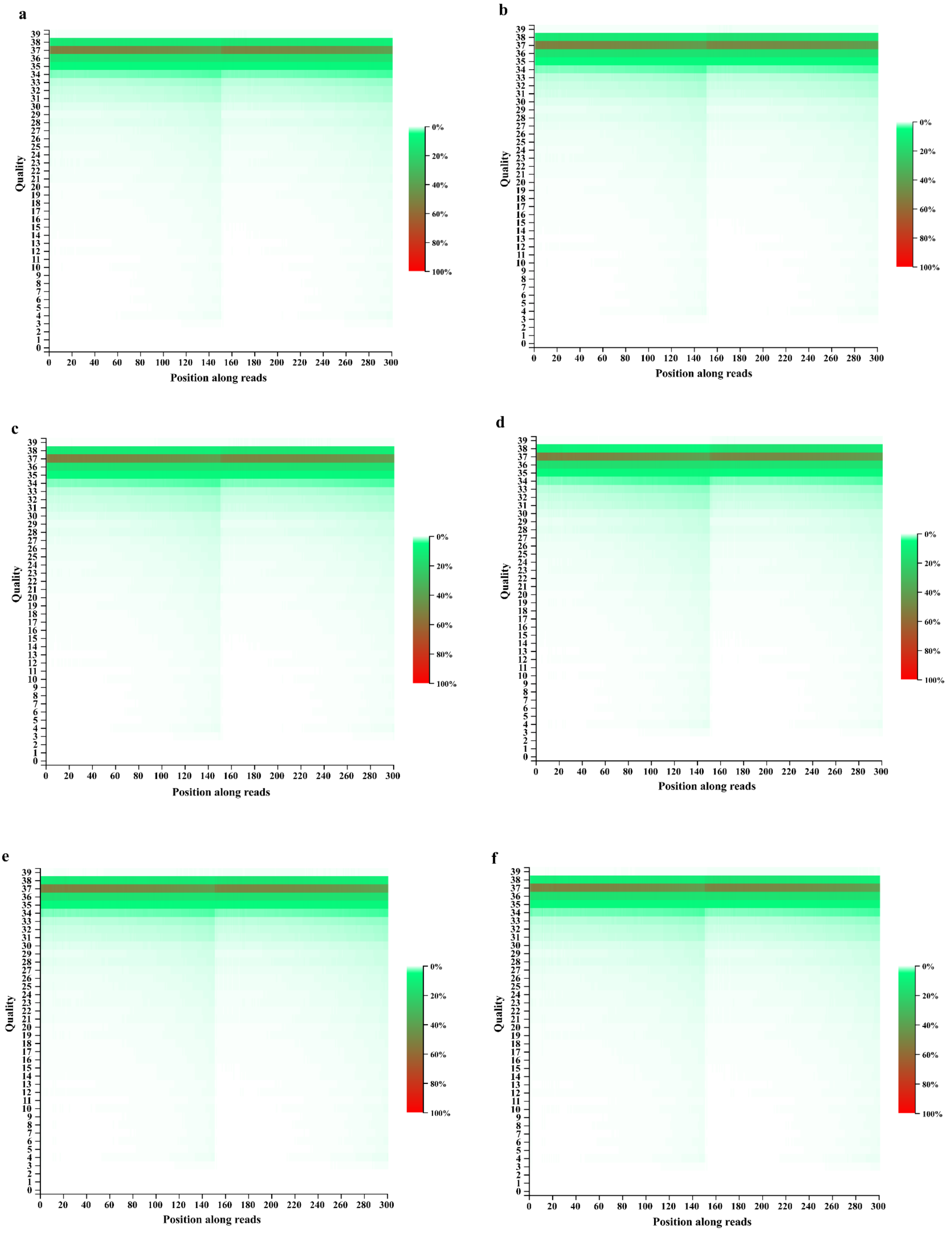
a
Quality
Position along reads
0%
20%
40%
60%
80%
100%
b
Quality
Position along reads
0%
20%
40%
60%
80%
100%
c
Quality
Position along reads
0%
20%
40%
60%
80%
100%
d
Quality
Position along reads
0%
20%
40%
60%
80%
100%
e
Quality
Position along reads
0%
20%
40%
60%
80%
100%
f
Quality
Position along reads
0%
20%
40%
60%
80%
100%

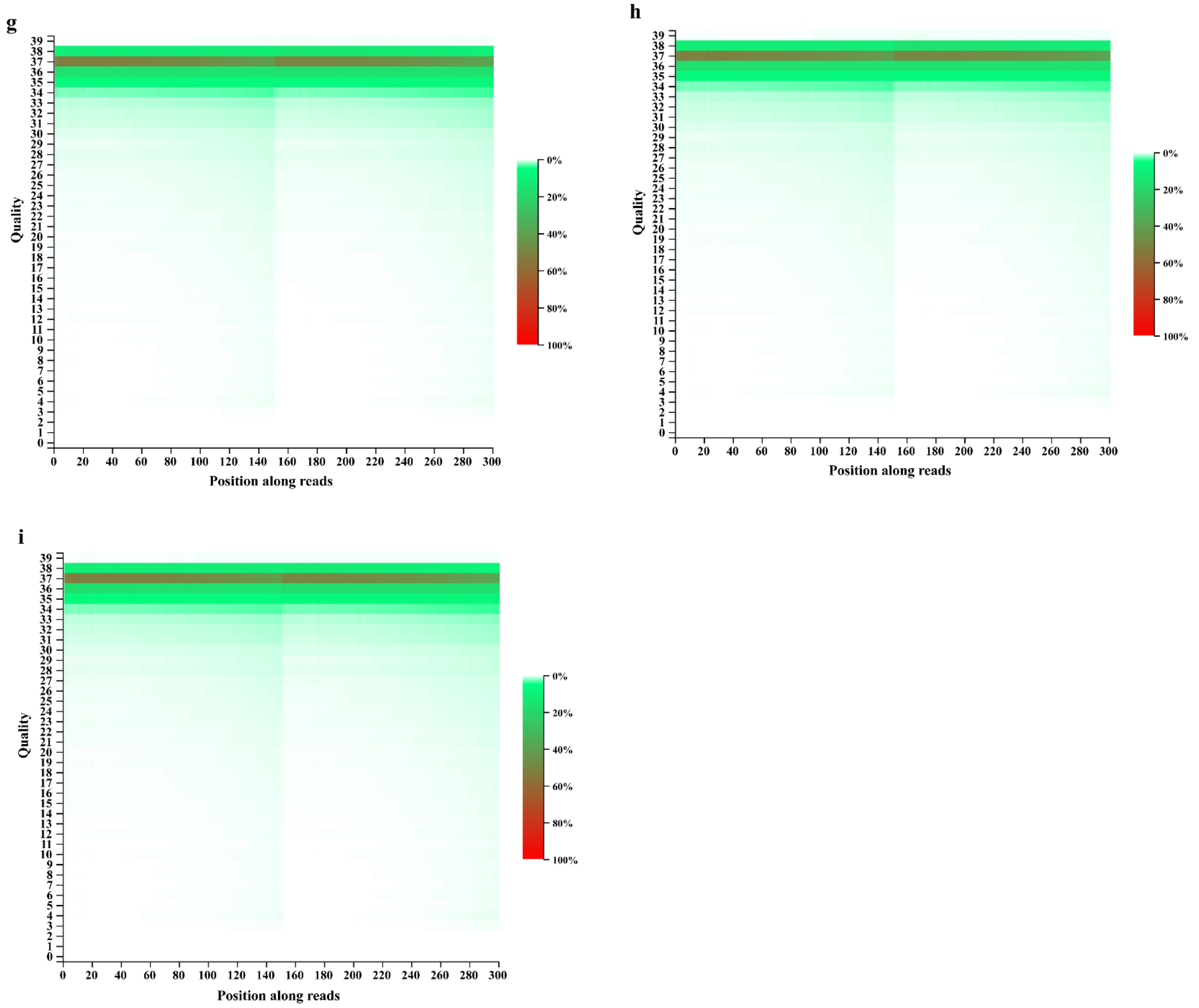

Figure S1. Base quality analysis of clean reads for sequencing library of LCK_a (a), LCK_b (b), LCK_c (c), LT1_a (d), LT1_b (e), LT1_c (f), LT2_a (g), LT2_b (h) and LT2_c (i). LCK, control; LT1, salinity treatments for 1 d; LT2, salinity treatments for 28 d. a, b, c represent the three repetitions.

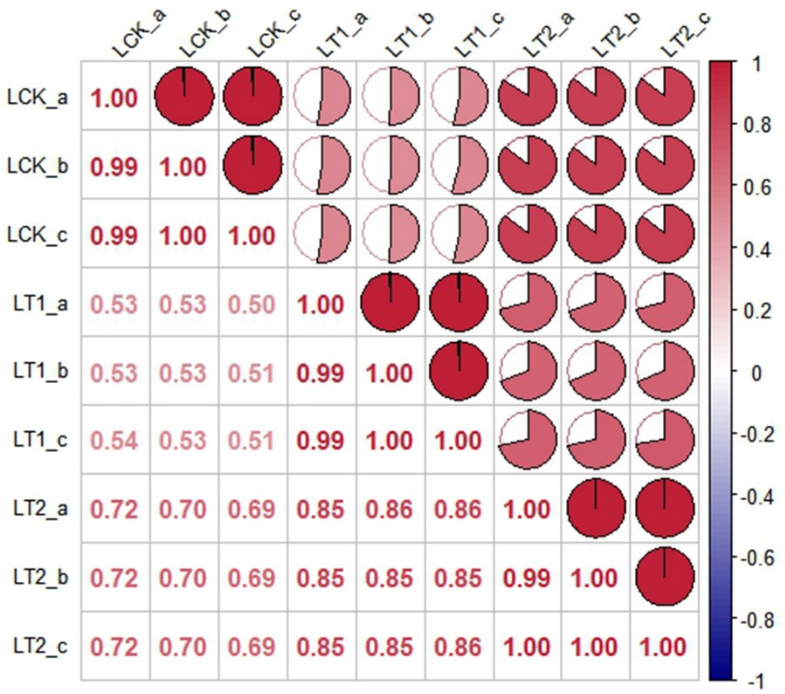

Figure S2. Pearson correlation analysis between samples based on gene expression profiles. LCK, control; LT1, salinity treatments for 1 d; LT2, salinity treatments for 28 d. a, b, c represent the three repetitions.

Table S1. The primer sequences used for quantitative real-time PCR (qRT-PCR) analysis.

**Gene ID**	**Forward primer（5′ →3′ ）**	**Reverse primer （5′ →3′ ）**
isoform_290716	AGCTAGATAACAAGAATGAT	TTATCATTCTTGTTATCTAGC
isoform_82715	CGGTGCTCCTCCTCCTCTTCTG	GAGGCCATCAAAGGCAGAGG
isoform_22449	ACCTATCTATCAGGAGGAATC	CTTCCGAGTCTCTTCCGTCA
isoform_49093	ATTCGCCGAGAAACCGTTCCA	ATTCGCCGAGAAACCGTTCCAC
isoform_158609	TAAGCAGTGGTATCAACGCA	CTCTGCGTTGATACCACTGCTTA
isoform_37195	ATGCTTTCCGTTTGGCACT	AGAGGCGAAGTGCCAAACGGA
18s rRNA	CCGCCTCTGGTGTGCACCGGTC	CCCCCGGAACCCAAGGACT
