# Supplementary figures and images for: PacBio Full-Length Transcriptome Sequencing Reveals the Mechanism of Salt Stress Response in Sonneratia apetala
Source: Plants (Basel). 2023 Nov 14;12(22):3849. doi: 10.3390/plants12223849 (PMC10675792; doi:10.3390/plants12223849)

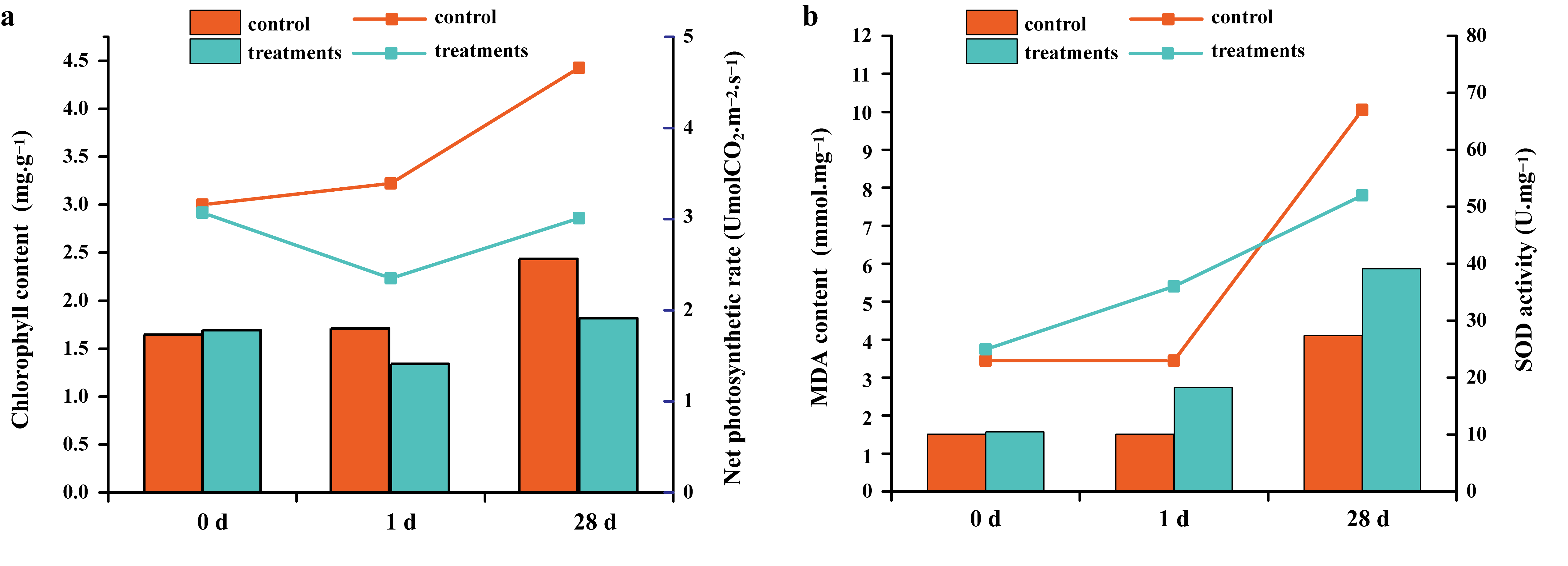

Supplement: Supplementary file 1 [file plants-12-03849-s001.zip › Figures and Supplementary Files/Figures/Figure 1.png]

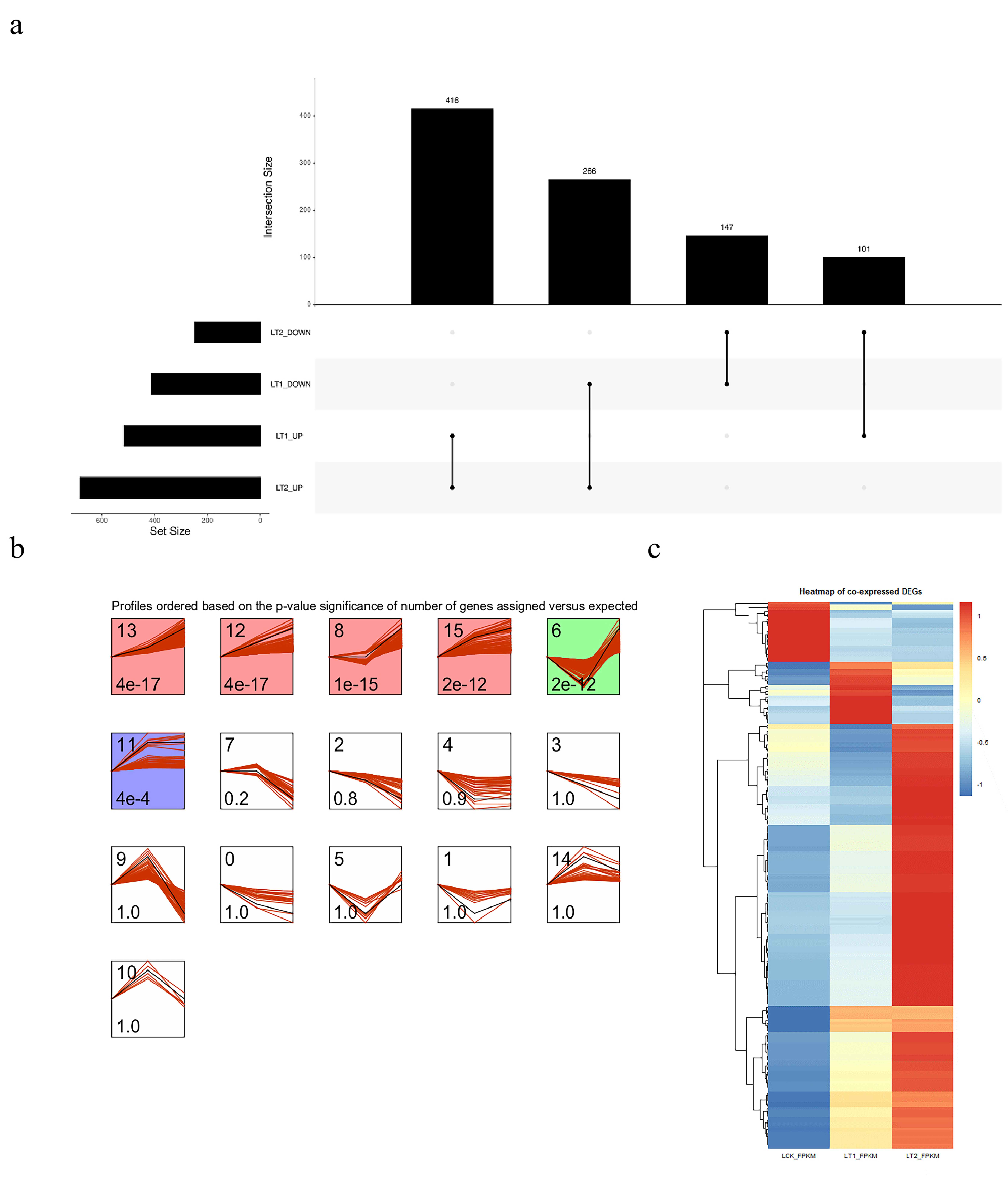

Supplement: Supplementary file 1 [file plants-12-03849-s001.zip › Figures and Supplementary Files/Figures/Figure 2.jpg]

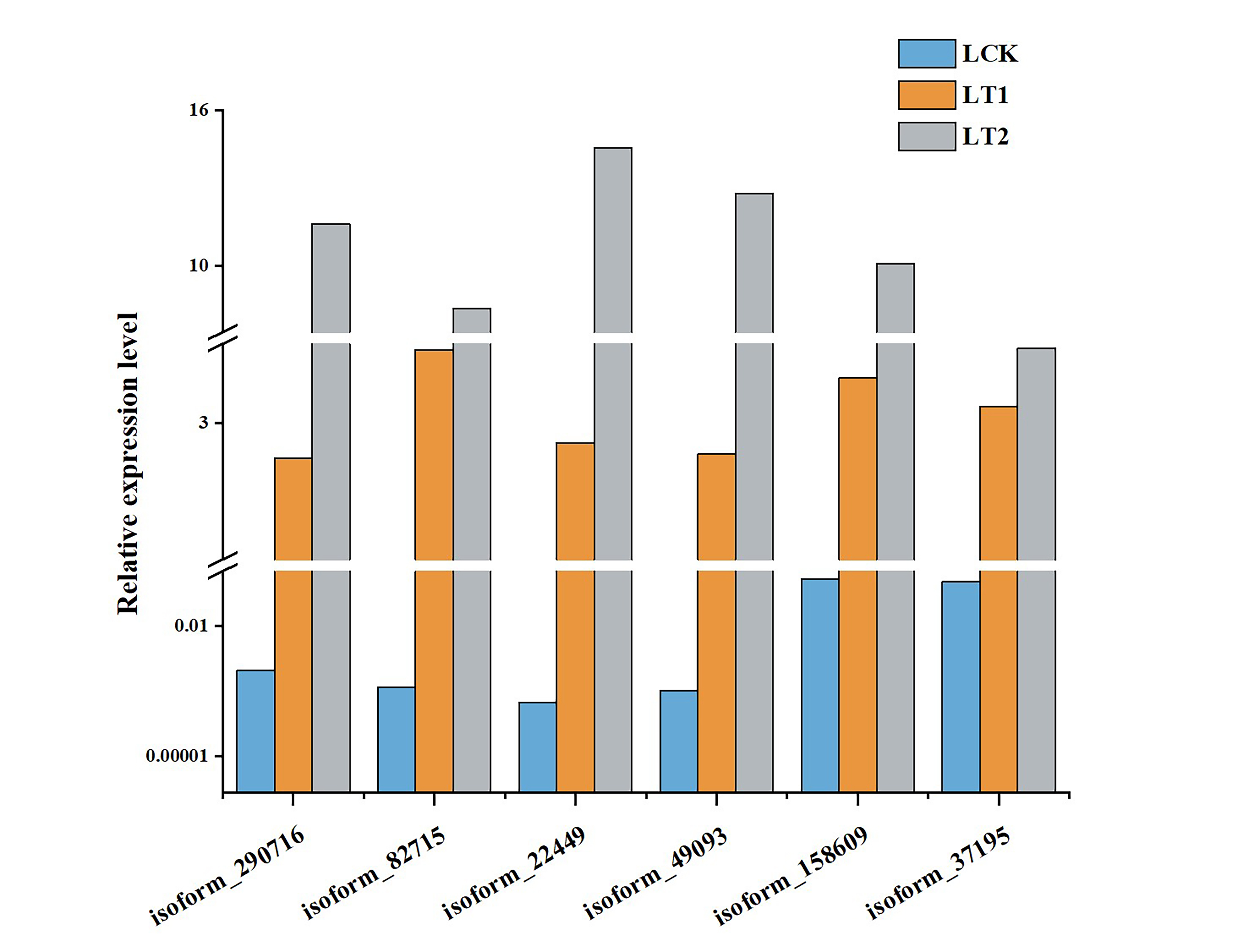

Supplement: Supplementary file 1 [file plants-12-03849-s001.zip › Figures and Supplementary Files/Figures/Figure 3.jpg]

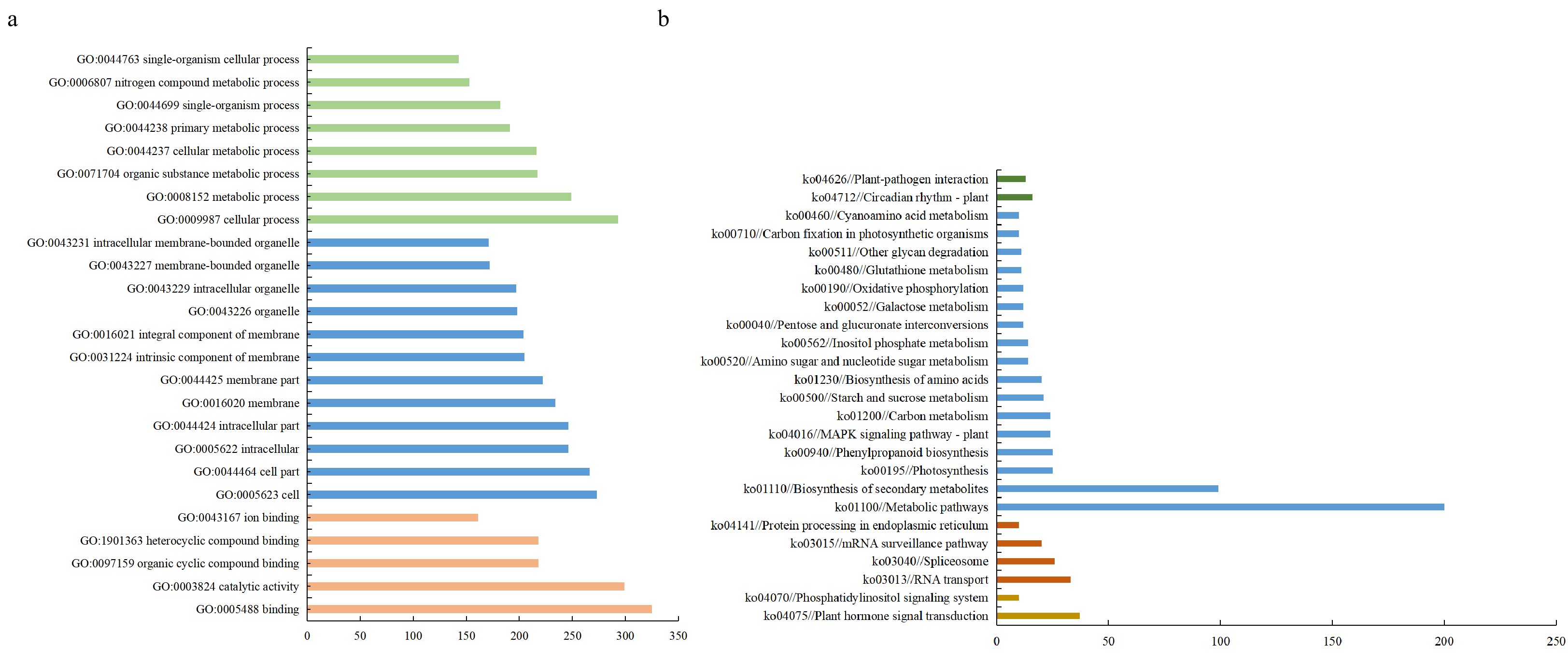

Supplement: Supplementary file 1 [file plants-12-03849-s001.zip › Figures and Supplementary Files/Figures/Figure 4.jpg]

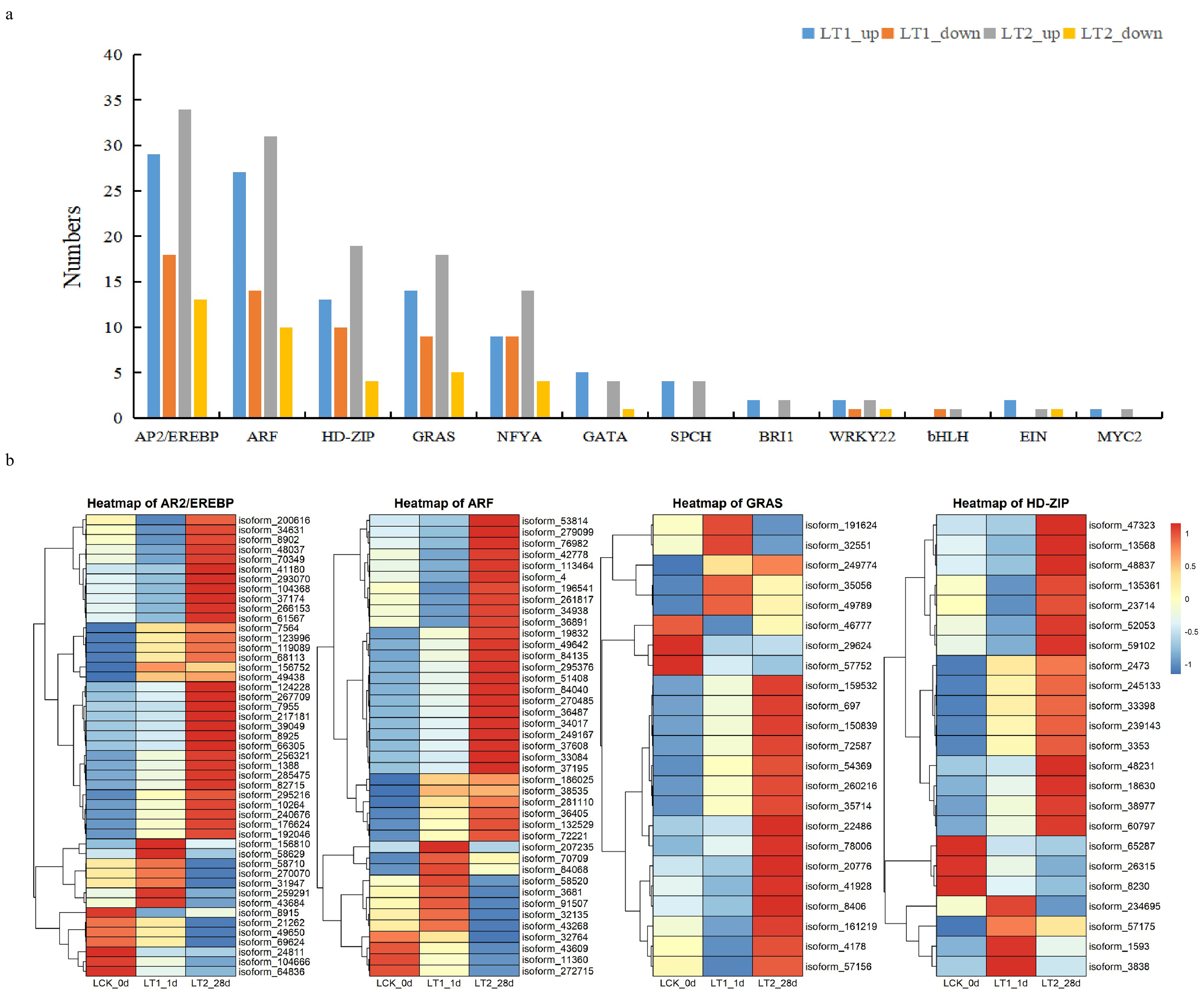

Supplement: Supplementary file 1 [file plants-12-03849-s001.zip › Figures and Supplementary Files/Figures/Figure 5.jpg]

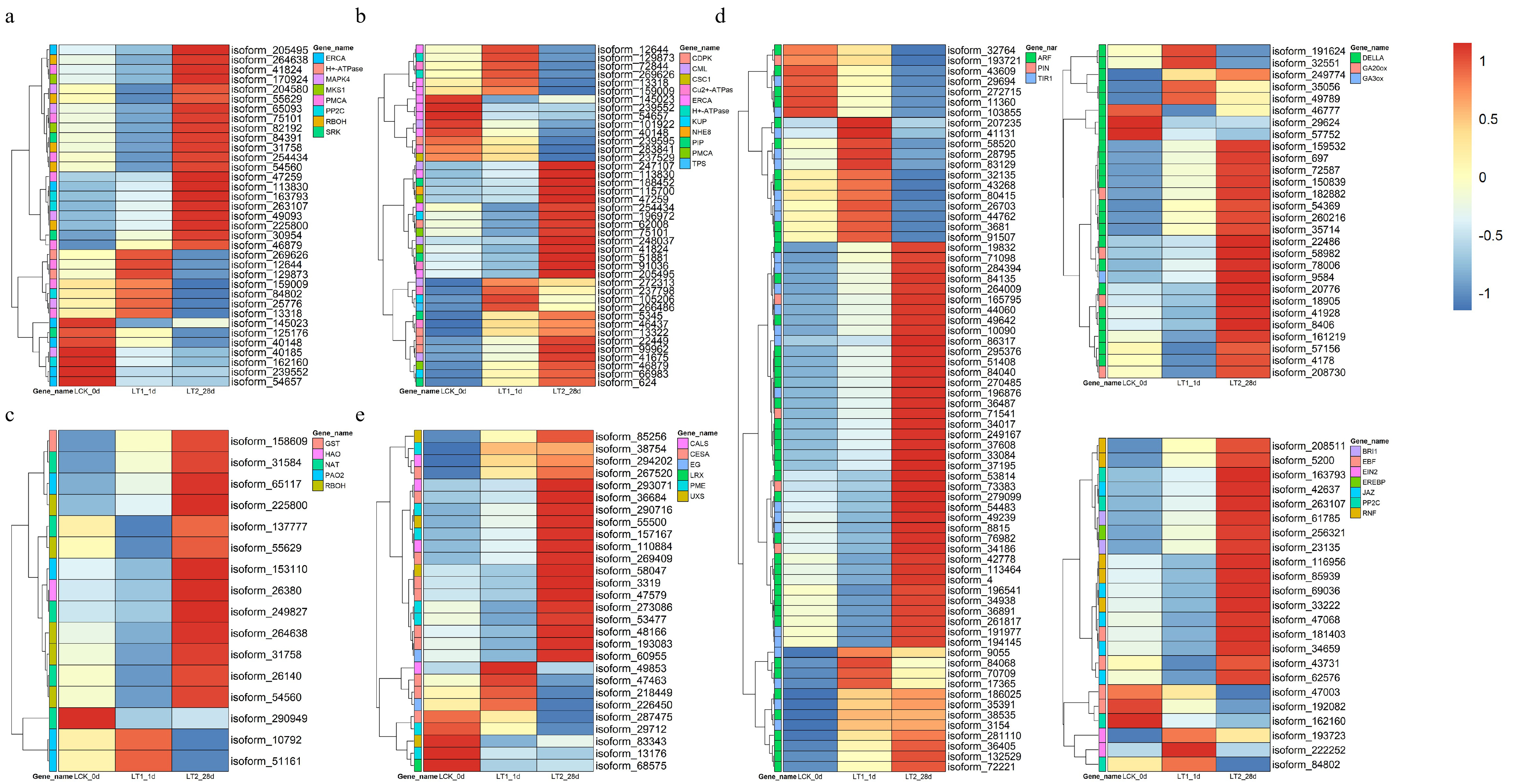

Supplement: Supplementary file 1 [file plants-12-03849-s001.zip › Figures and Supplementary Files/Figures/Figure 6.jpg]

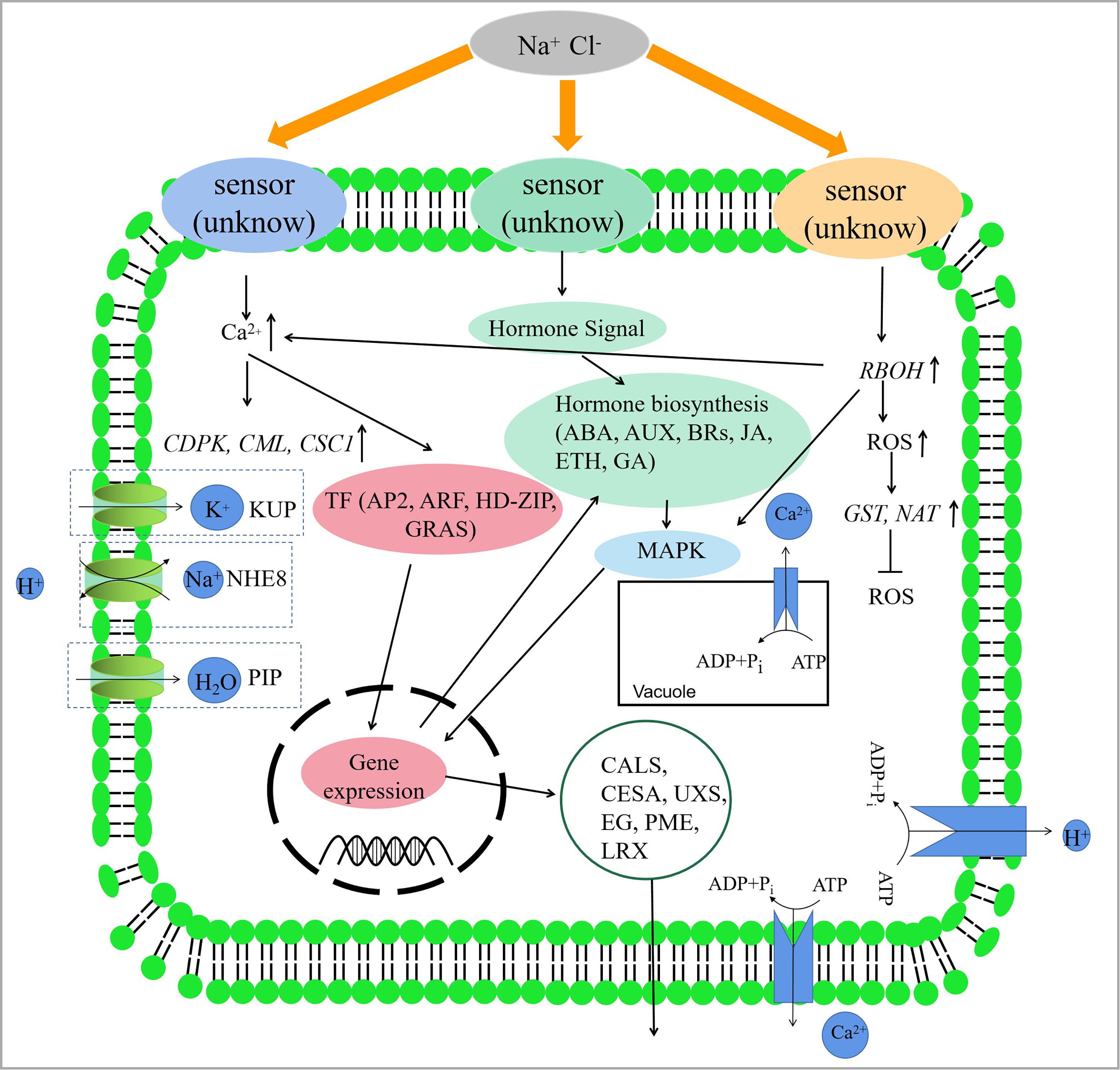

Supplement: Supplementary file 1 [file plants-12-03849-s001.zip › Figures and Supplementary Files/Figures/Figure 7.jpg]
